# White Matter Abnormalities in the Corpus Callosum in Acute and Recovered Anorexia Nervosa Patients—A Diffusion Tensor Imaging Study

**DOI:** 10.3389/fpsyt.2019.00490

**Published:** 2019-07-08

**Authors:** Kathrin Nickel, Ludger Tebartz van Elst, Lukas Holovics, Bernd Feige, Volkmar Glauche, Tina Fortenbacher, Dominique Endres, Almut Zeeck, Oliver Tüscher, Andreas Joos, Simon Maier

**Affiliations:** ^1^Department of Psychiatry and Psychotherapy, Medical Center - University of Freiburg, Faculty of Medicine, University of Freiburg, Freiburg, Germany; ^2^Department of Psychosomatic Medicine and Psychotherapy, Medical Center - University of Freiburg, Faculty of Medicine, University of Freiburg, Freiburg, Germany; ^3^Department of Neurology, Medical Center - University of Freiburg, Faculty of Medicine, University of Freiburg, Freiburg, Germany; ^4^Department of Psychiatry and Psychotherapy, University of Mainz, Mainz, Germany; ^5^Department of Psychotherapeutic Neurology, Kliniken Schmieder, Gailingen, Germany

**Keywords:** anorexia nervosa, diffusion tensor imaging, fractional anisotropy, mean diffusivity, corpus callosum

## Abstract

**Objective:** Severe malnutrition in patients with anorexia nervosa (AN) as well as possible trait-related aberrations lead to pronounced structural brain changes whose reversibility after recovery is currently unclear. Previous diffusion tensor imaging (DTI) studies investigating white matter (WM) microstructure alterations in AN are inconsistent.

**Methods:** In this so far largest DTI study in adults, we investigated 33 AN patients, 20 recovered (REC), and 33 healthy women. DTI data were processed using the “DTI and Fiber tools,” and the Computational Anatomy Toolbox. WM integrity, both in terms of fractional anisotropy (FA) and mean diffusivity (MD), was assessed.

**Results:** We found a significant FA decrease in the corpus callosum (body) and an MD decrease in the posterior thalamic radiation in the AN group. The REC group displayed FA decrease in the corpus callosum in comparison to HC, whereas there were no MD differences between the REC and HC groups.

**Conclusion:** Despite prolonged restoration of weight in the REC group, no significant regeneration of WM integrity in terms of FA could be observed. Transient changes in MD likely represent a reversible consequence of the acute state of starvation or result from dehydration. Reduction of FA either may be due to WM damage resulting from malnutrition or may be considered a pre-morbid marker.

## Introduction

Anorexia nervosa (AN) is a severe mental disorder associated with persistent restriction of energy intake leading to a significantly low body weight, a preoccupation with weight gain, and an altered body perception (1).

Pathophysiology is currently unclear, but many studies point toward the involvement of various interacting developmental, genetic, environmental, and neurobiological factors (2, 3).

In terms of neurobiological alterations, most previous structural imaging studies focused on volumetric gray matter (GM) or white matter (WM) alterations and mainly reported reductions of these two measures in acute AN (4–6). A growing number of studies investigated WM integrity in AN performing diffusion tensor imaging (DTI). DTI is a noninvasive imaging technique that allows quantitative maps of microscopic, natural displacements of water molecules that occur in brain tissues as part of physical diffusion processes (7). Fractional anisotropy (FA) as an imaging marker is a scalar value of the degree of anisotropic/directional diffusion within a voxel (8). FA is linked to axon diameter, membrane permeability, and myelination, as well as packing density of fibers (9). Lower FA reflects isotropic, i.e., either unrestricted or equally restricted diffusion in all spatial directions (10). Another important marker is mean diffusivity (MD) defined as the average diffusion irrespective of directionality (8). MD is a sensitive marker that can be altered by any disease process that affects the barriers (e.g., cell membranes) which restrict water diffusion (11). Increased tissue water in edema was reported to increase, whereas cell proliferation in neoplasia may decrease MD (8).

**Table 1** displays previous DTI investigations in AN. Several studies reported WM alterations in AN in comparison to healthy controls (HC). Nevertheless, the direction and localization of abnormalities is inconsistent with some studies suggesting increased (17, 19, 22, 25), others decreased (12, 14, 15, 21–25, 27, 28), FA in acute AN. A recent study with a modest sample of adolescents with AN detected no WM microstructure alterations [FA, MD, radial diffusivity (RD: magnitude of water molecule displacements perpendicular to WM pathways), axial diffusivity (AD: rate of diffusion in the parallel direction (18)]. Other studies investigating MD, RD, or AD also found inconsistent results with some reporting decreased MD, RD, or AD in different brain areas (15, 17, 21, 24), others increased (21, 23–25, 28) or no alterations (15, 17, 18) in AN. Discrepant findings may result from differences in age group composition, disease duration, in applied analysis methods and definition of inclusion criteria. With respect to age effects, WM differences were reported in both adolescents and adults with AN. Yet, those findings are inconsistent. However, it remains to be investigated whether longer duration of illness or an early onset during adolescence might facilitate WM defects.

**Table 1:** Previous diffusion tensor imaging studies in anorexia nervosa.

Study	n (AN/REC/HC)	Age in years (mean ± SD)	Methods	Region(s) and results (AN/REC vs HC)
**1. Von Schwanenflug et al., 2018 (** 12 **)**	56 AN 44 REC 60 HC	15.86 ± 2.93 15.40 ± 2.28 15.64 ± 2.27 16.19 ± 2.89	TBSS	FA↓: body corpus callosum in AN vs HC No differences between REC and HC Longitudinal: FA↑ with weight gain in REC relative to AN in large parts of body corpus callosum and fornix; FA↓ in right corticospinal tract
**2. Bang et al., 2018 (** 13 **)**	21 REC (for 1year) 21 HC	27.62 ± 5.06 26.10 ± 4.75	FSL TBSS	No differences in WM microstructure (FA, AD, MD and RD) between REC and HC
**3. Hu et al., 2017 (ᆔ** 14 **)**	8 AN 14 HC	17.6 ± 2.2 19.1 ± 3.1	SPM voxel-based method	FA↓: left superior frontal gyrus, medial frontal gyrus, anterior cingulate cortex, middle frontal gyrus, inferior frontal gyrus, thalamus, bilateral insula
**4. Gaudio et al., 2017 (** 15 **)**	14 AN 15 HC	15.7 ± 1.6 16.3 ± 1.5	FSL TBSS	FA↓: left anterior and superior corona radiata, left superior longitudinal fasciculus (SLF), fornix, body corpus callosum AD↓: SLF bilaterally, left superior and anterior corona radiata, and external capsule, right posterior limb of the internal capsule, right posterior thalamic radiation RD, MD: no significant differences
**5. Zhang et al., 2016 (** 16 **)**	24 REC 31 HC 29 BDD	21.3 ± 4.5 20.9 ± 3.9 23.2 ± 5	DTI Studio whole-brain WM tractography	AN showed abnormal network modularity involving frontal, basal ganglia, and posterior cingulate nodes No standard analysis of FA, MD, AD, RD
**6. Vogel et al., 2016 (ᆔ** 17 **)**	22 AN 9 REC 21 TD	15.0 ± 1.6 14.8 ± 2.3 15.2 ± 1.3	FSL TBSS	FA↑: bilateral superior region of corona radiata, corpus callosum, anterior and posterior thalamic radiation, anterior and posterior limb of internal capsule, left inferior longitudinal fasciculus Elevated FA at admission was associated with reduced MD and RD, but not AD FA↑: partially normalized after weight rehabilitation
**7. Pfuhl et al., 2016 (ᆔ** 18 **)**	35 AN 32 REC 62 HC	16.1 ± 2.8 22.5 ± 3.0 16.4 ± 2.0	FSL, TRACULA	No group differences in FA, MD, RD, AD after correction for multiple comparisons.
**8. Cha et al., 2016 (** 19 **)**	22 AN 18 HC	19.5± 2.4 20.5 ± 3.0	FSL, TBSS	FA↑: fronto-accumbal WM ROI near the lateral orbitofrontal cortex and nucleus accumbens both before and after weight restoration
**9. Shott et al., 2016 (** 20 **)**	24 REC 24 HC	30.3 ± 8.1 27.4 ± 6.3	FSL, TBSS	REC FA↓: anterior corona radiata, capsula interna, cerebellum (corticopontine tract, inferior, and middle peduncle), corpus callosum, anterior thalamic radiation, inferior fronto-occipital, uncinate fasciculus MD, RD, AD: no differences
**10. Hayes et al., 2015 (** 21 **)**	8 AN 8 TD	35.0 ± 11.0 36.0 ± 9.0	FSL, 3D Slicer	FA↓: bilateral anterior limb of capsula interna, right anterior cingulum, left inferior fronto-occipital fasciculus, left crus fornix AD↓: right anterior limb of capsula interna, right anterior cingulum RD↑: bilateral anterior limb of capsula interna, left inferior fronto-occipital fasciculus, left crus fornix
**11. Travis et al., 2015 (** 22 **)**	15 AN 15 HC	16.6 ± 1.4 17.1 ± 1.3	mrDiffusion	FA↓: right anterior superior longitudinal fasciculus, bilateral fibria-fornix, corpus callosum FA↑: right anterior thalamic radiation, left anterior SLF
**12. Via et al., 2014 (** 23 **)**	19 AN 19 TD	28.37 ± 9.55; 28.63 ± 8.58	FSL, TBSS	FA↓: parietal part of the left SLF and the fornix MD, RD↑: SLF and fornix AD↑: fornix
**13. Nagahara et al., 2014 (ᆔ** 24 **)**	17 AN 18 TD	23.8 ± 6.68 26.2 ± 5.6	FSL, TBSS	FA↓: left cerebellar hemisphere MD↑: anterior body of the fornix MD↓: right corpus callosum, right SLF
**14. Frank et al., 2013 (ᆔ** 25 **)**	19 AN 22 TD	15.4 ± 1.4 14.8 ± 1.8	SPM, NordicICE	FA↓: left fornix, bilateral cingulum, right forceps major, right superior, and left posterior corona radiate, occipital part corpus callosum FA ↑: left SLF, bilateral anterior corona radiata, bilateral inferior fronto-occipital fasciculus ADC↑: left fornix, right corpus callosum, right corticospinal tract, right posterior corona radiata, bilateral corticopontine tract, bilateral SLF
**15. Yau et al., 2013 (** 26 **)**	12 REC 10 TD	28.7 ± 7.9 26.7 ± 5.4	FSL	FA: No group differences MD↓, AD, and/or RD↓: left superior frontal WM including corona radiata (superior and posterior), corpus callosum (body and bilateral splenium), posterior limb of capsula interna, left SLF, left posterior cingulum, precuneus, superior parietal WM, left dorsal cingulum, right precuneus, and posterior corona radiata, right posterior cingulum, and posterior corona radiata
**16. Frieling et al., 2012 (** 27 **)**	12 AN 9 REC 20 TD	26.8 ± 6.9 27.4 ± 5.3 24.8 ± 2.6	SPM	FA↓ (AN and REC): bilateral posterior thalamic radiation (optic radiation, left mediodorsal thalamus), bilateral posterior corona radiate, left middle cerebellar peduncle, parts of left SLF
**17. Kazlouski et al., 2011 (ꚤ** 28 **)**	16 AN 17 TD	24 ± 7 25 ± 4	SPM, DTI Studio	FA↓: bilateral fimbria-fornix, fronto-occipital fasciculus, posterior cingulum WM

AN, anorexia nervosa; REC, recovered; HC, healthy controls; SD, standard deviation; WM, white matter; FA, fractional anisotropy; MD, mean diffusivity; RD, radial diffusivity; AD, axial diffusivity; ADC, apparent diffusion coefficient; DTI, diffusion tensor imaging; TBSS, tract-based spatial statistics; SPM, statistical parametric mapping; TRACULA, TRActs Constrained by UnderLying Anatomy; SLF, superior longitudinal fasciculus; ROI, region of interest.

Further, methodological differences between tract-based spatial statistics (TBSS) and voxel-based analysis (VBA) could account for heterogeneity in WM findings, as TBSS seems to be more sensitive to FA reductions (29). Concerning body mass index (BMI) differences, an interesting study of Olivo et al. (30) compared 25 adolescents with atypical AN with 25 HC, who did not differ with respect to BMI. They did not find WM differences and discussed weight-related WM abnormalities as most likely in other studies. However, it might also reflect a sample characterized by missing tendencies to lose weight (and no premorbid WM abnormalities). Furthermore, disease duration was very short in this sample.

Various areas of altered microstructural integrity in AN have been described. A recent meta-analysis (n = 13) reported decreased FA in the corpus callosum, the left superior longitudinal fasciculus II, the left precentral gyrus, as well as increased FA in the right cortico-spinal projections, and lingual gyrus in AN in comparison to HC (31). Additionally, altered WM integrity was reported in the corona radiata (25, 27, 32), the fornix (21–22, 23, 25, 28, 32), and the cingulum (14, 21, 25, 28).

The DTI studies on individuals recovered from AN (REC) are scarce with inconsistent findings of cross-sectional investigations reporting either no differences in microstructural integrity between REC and HC (13, 18, 26), or FA decrease in REC (20, 27). A limited number of longitudinal studies reported only partial normalization after weight rehabilitation (17, 19), whereas others proposed complete reversibility (12). Since there are only a few studies to date that examine REC subjects, meta-analyses have not been able to carry out subgroup analyses (31).

## Aims of the Study

Based on the available evidence, we aimed to identify brain regions with WM microstructural abnormalities in acute AN. Furthermore, we intended to obtain insight into whether there are WM alterations in the REC in comparison to the HC group. Regarding the heterogeneous result pattern concerning FA and MD, we tested for increase, as well as decrease, of these signals. Given the strict definition of recovery in our sample (see Materials and Methods), we hypothesized no differences in WM microstructure (FA, MD) between the REC and HC groups. As different areas of WM microstructure alterations in AN have been described, we did not restrict our analysis to *a priori* regions of interest (ROIs).

## Materials and Methods

### Participants

The present study was approved by the ethics committee of the University Medical Center Freiburg (Approval ID: 520/13). Patients were recruited from the Department of Psychosomatic Medicine and Psychotherapy of the University Medical Center Freiburg. Thirty-three adult women with AN, 20 participants with a previous history and current recovery of AN (REC) and 33 HC were included in the study. Following written informed consent, magnetic resonance imaging (MRI) scans were obtained.

Inclusion criteria for the AN group were a BMI ≤ 18.5 kg/m² and an age of ≥ 18 years. AN was diagnosed by senior consultants according to the DSM-5 criteria. Furthermore, an in-depth evaluation including the Eating Disorder Examination Interview (EDE) (33) was performed. Twenty-nine AN patients were of the restrictive subtype, whereas four were of the binge-eating/purging subtype. Most AN participants were recruited *via* our outpatient department (25 AN), whereas eight patients had just started the inpatient treatment. Outpatients were offered inpatient multimodal psychotherapeutic treatment comprising cognitive behavioral therapy, as well as systemic and psychodynamic modalities.

For inclusion in the REC group, participants did not suffer from eating problems for at least 1 year prior to scanning. Further, a minimum BMI ≥ 20 kg/m^2^ was set. Most, but not all REC met this criterion. Four participants had a BMI slightly below 20 kg/m^2^ (19.3−19.8 kg/m^2^) and 2 a BMI of 18.5−19.0 kg/m^2^. These participants had not exceeded a BMI of this range previous to the onset of the disorder and were clinically completely recovered. The latter was tested using the EDE (33), and scores had to be within one standard deviation of normal, which is a strict criterion. Nineteen REC were of the restrictive, whereas one was of the binge eating/purging subtype.

The AN, REC, and HC participants were matched, with respect to the intelligence quotient (IQ). They were standardized with regard to hormone status (all participants were amenorrheal or in the luteal phase of the menstruation cycle at the scanning date (REC, HC); if taking oral contraceptives, they had to be in phase when taking both progesterone and estrogen, i.e., similar to the luteal phase).

The following psychometric tools were used in all participants: SKID I, SKID II (34), Beck Depression Inventory (BDI-II) (35, 36), EDE (33), Eating Disorder Inventory (37), and the State-Trait Anxiety Inventory (STAI) (38). IQ was assessed with the Multiple-Choice Word Test B (MWT-B) (39) as a measure of estimated premorbid IQ.

We defined the following exclusion criteria for all three groups: schizophrenia, bipolar I disorder, a history of neurological diseases, substance abuse, a severe medical illness or general contraindications for MRI (claustrophobia, metallic implants, pregnancy). No participant took any psychiatric medication, except one AN patient who had just started escitalopram but had not yet reached an effective serum level.

The analysis of GM and WM volumes as well as of cortical thickness of an overlapping sample has already been published elsewhere (40). From the sample reported by Nickel et al. (40), three AN had to be excluded due to spiking artifacts in the DTI data, whereas two AN with minor head motion artifacts were only excluded in the voxel-based morphometry analysis. In four HC, no DTI data were recorded (termination of the measurement by the participant) and four had spiking or head motion artifacts. In the REC sample, three subjects were excluded due to spiking artifacts and of one subject no DTI sequence was recorded.

### Image Acquisition

Image acquisition of all participants took place between March 2015 and April 2017. Scanning was performed with a 3T Siemens PRISMA Magnetom (Erlangen, Germany) equipped with a 20-channel head coil for signal reception. A standard MPRAGE (magnetization-prepared rapid gradient echo) T1-weighted anatomical scan was obtained for each participant with the following parameters: relaxation time = 2,300 ms, echo time = 2.98 ms, flip angle = 9°, field of view (FOV) = 240 × 256 mm^2^, voxel size = 1 × 1 × 1 mm^3^. We used a single-shot, spin echo, echo planar (EPI) sequence to obtain diffusion weighted images for each participant. For the calculation of the diffusion tensor, 61 spatial directions were respected. The b-value for control of diffusion weighting was set at 1,000 s/mm^2^. We chose the imaging parameters for the DTI sequence as follows: FOV = 192 × 192 mm^2^, slices = 60, echo time = 80 ms, voxel size = 2 × 2 × 2 mm^3^.

### Preprocessing

All EPI images were corrected with a reliable and fully automated distortion correction (41). Before data analysis, all DTI images were screened carefully for motion or spike artifacts using the SPM Artrepair toolbox.

### Processing

The diffusion tensor was calculated with the software “DTI and Fiber Tools” (42). Diffusion in 61 spatial directions was registered for tensor calculation. The FA and the MD values of each voxel were computed from corresponding diffusion tensors.

With SPM12 [Statistical Parametric Mapping Software, Wellcome Department of Cognitive Neurology, University College London UK; for details, see Ref. (43)] in Matlab R2012 (Mathworks, Sherborn, MA), we performed a co-registration of anatomical MPRAGE images onto the B0-images. Segmentation and normalization of MPRAGE images was carried out with Computational Anatomy Toolbox (http://dbm.neuro.uni-jena.de/vbm.html). After segmentation procedure, GM, WM, and cerebrospinal fluid (CSF) segments were available. For the normalization into MNI space, the deformation fields derived from the normalization step were applied to the individual FA and MD maps. Smoothing was performed with an 8-mm full width at half-maximum Gaussian kernel.

### Statistical Analysis

#### Psychometric Data

Group comparisons of demographic and psychometric data (age, IQ, psychometric scores) were carried out using SPSS software, version 22 (IBM Corp., Armonk, NY). We conducted an analysis of variance (ANOVA) followed by a *post hoc* Tukey-Kramer Test.

#### Analysis of DTI Data

Analysis of imaging data was performed in SPM12 and Matlab R2012 (Mathworks, Sherborn, MA). We calculated group-wise comparisons (AN versus HC, REC versus HC and vice versa) applying SPM-t-contrasts. Age and total intracranial volume (TIV) were respected as covariates to exclude confounding effects. We applied a statistical threshold of p < 0.05 after family-wise error (FWE) correction.

In a further analysis, the BDI-II (35, 36) was added as a covariate to correct for the influence of depressiveness.

#### Regression Models

Additionally, we run SPM regression models of FA and MD values with the EDE total score (33) and BMI across all groups.

## Results

### Demographic and Psychometric Data

**Table 2** lists the demographic and psychometric data of the AN, the REC, and the HC group. For final data analysis, 33 patients with AN, 20 participants with a previous history of AN currently recovered (REC), and 33 HC were included. The three groups showed no significant differences concerning gender or IQ according to the MWT-B (39) (Table 2). As expected, the REC sample was older than the AN or HC samples. The AN participants showed a lower BMI and scored higher in the BDI-II (35, 36) and STAI (38) questionnaire than HC. According to SKID I (34), seven AN were diagnosed with a current major depression, two AN and one REC participant with a specific phobia, and two REC with a social anxiety disorder.

**Table 2:** Demographic and psychometric data.

	AN *(n=33)* mean ± SD	REC *(n=20)* mean ± SD	HC (*n*=33) mean ± SD	ANOVA	Post-hoc Tukey-Kramer test
**Age (y)**	24.3 ± 4.2	27.2 ± 7.4	23.0 ± 2.5	df = 2,83; F = 5.2; p = 0.007	REC>AN, HC
**Current BMI (kg/m²)**	16.3 ± 1.4	20.7 ± 1.3	21.9 ± 2.3	df = 2,83; F = 88.8; p < 0.001	HC>REC>AN
**Illness duration (y)**	6.7 ± 3.8	5.5 ± 5.1	Not applicable		
**Lowest lifetime BMI (kg/m²)**	14.8 ± 1.4	14.6 ± 2.2	20.3 ± 1.7	df = 2,62; F = 58.3; p < 0.001	HC>REC, AN
**Duration of recovery**	Not applicable	4.5	Not applicable		
**EDE – total score**	3.2 ± 1.1	0.6 ± 0.4	0.4 ± 0.3	df = 2,83; F = 142.8; p < 0.001	AN>REC, HC
**EDI-2 – total score**	61.1 ± 8.9	47.3 ± 5.1	44.2 ± 3.0	df = 2,83; F = 65.7; p < 0.001	AN>REC, HC
**BDI-II**	20.9 ± 10.6	6.0 ± 6.1	2.2 ± 2.9	df = 2,83; F = 56.4; p < 0.001	AN>REC, HC
**STAI – State Score**	39.0 ± 7.0	35.2 ± 5.5	32.4 ± 4.7	df = 2,83; F = 10.5; p < 0.001	AN, REC>HC
**STAI –Trait Score**	46.7 ± 7.5	31.4 ± 7.8	29.1 ± 6.8	df = 2,83; F = 53.8; p < 0.001	AN>REC, HC
**MWT-B**	28.5 ± 5.0	29.5 ± 5.0	27.7 ± 4.1	df = 2,83; F = 0.9; p = 0.4	
**Fasting before MRI (min)**	42.9 ± 10.0	49.3 ± 17.4	42.2 ± 12.4	df = 2, 81; F = 2.1; p = 0.1	
**Calories of food intake before scanning**	110.8 ± 114.4	290.5 ± 141.0	380.3±113.8	df = 2,83; F = 42.1; p < 0.001	HC>REC>AN

AN, anorexia nervosa; REC, recovered; HC, healthy controls; SD, standard deviation; y, years; min, minutes; ANOVA, analysis of variance; BMI, body mass index; EDE, Eating Disorder Examination Interview; EDI, Eating Disorder Inventory; BDI, Beck Depression Inventory; STAI, State-Trait-Anxiety Inventory; MWT-B, Multiple-Choice Word Test B; MRI, magnetic resonance imaging.

Twenty-nine AN (88%), 17 REC (85%), and 31 HC (94%) had successfully completed the highest school grade (“Abitur”) in the German school system.

### DTI Results

#### Fractional Anisotropy

**Figure 1:**
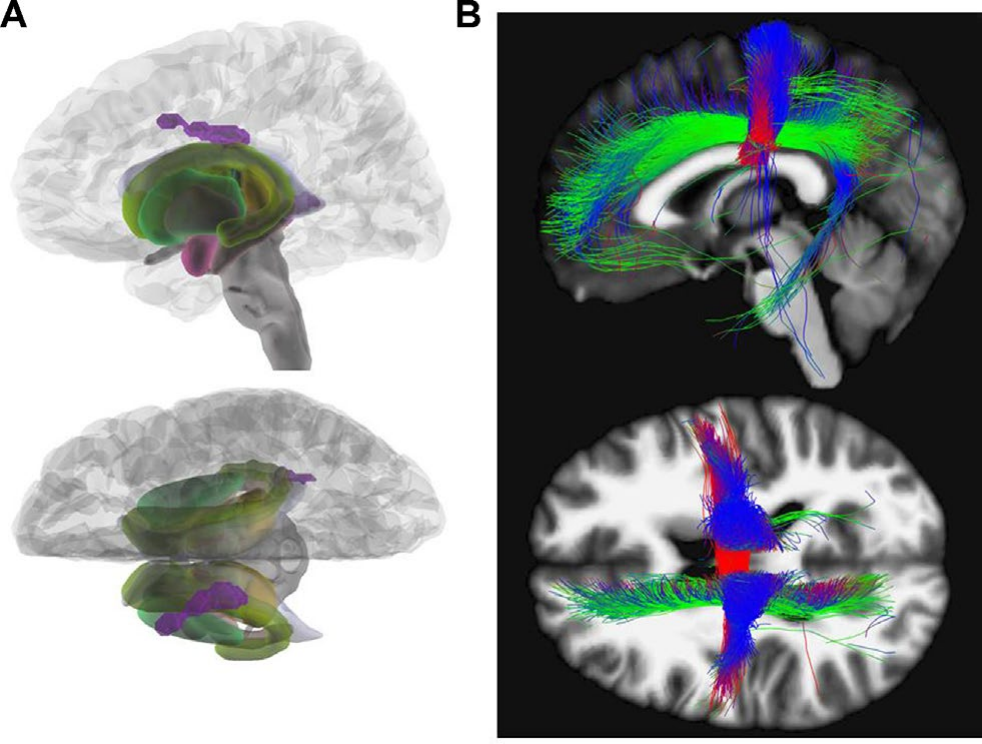
Fractional anisotropy. **(A)** Glass brain view of the difference in FA signal depicted in purple between AN and HC individuals in left and top view. Individuals with AN show significantly lower FA values in the body of the corpus callosum (x = −14, y = −15, z = 33; Z = 4.14) as compared to HC. **(B)** Network involvement of the region with abnormal FA signal. We tracked the connectivity of the ROI in the body of the corpus callosum (x = −14, y = −15, z = 33) which showed a significantly lower FA in AN to illustrate the network involvement. Red: left – right; green: anterior – posterior; blue: superior – inferior. FA, fractional anisotropy; AN, anorexia nervosa; HC, healthy controls; ROI, region of interest.

Calculating between group differences (HC versus AN), we found decreased FA in AN reaching significance on cluster level in the body of the corpus callosum (x = −14, y = −15, z = 33; Z = 4.14; k = 504; pFWEpeak = 0.219; pFWEcluster = 0.012). This difference was also observable in HC versus REC (x = −14, y = −15, z = 32; Z = 3.86; k = 368; pFWEpeak = 0.481; pFWEcluster = 0.040). After correction for effects of depression according to BDI-II (35, 36) only the HC versus REC contrast remained significant (x = −12, y = −15, z = 32; Z = 3.90; k = 369; pFWEpeak = 0.457; pFWEcluster = 0.039). **Figure 1** illustrates the results for FA contrasting AN versus HC.

#### Mean Diffusivity

**Figure 2:**
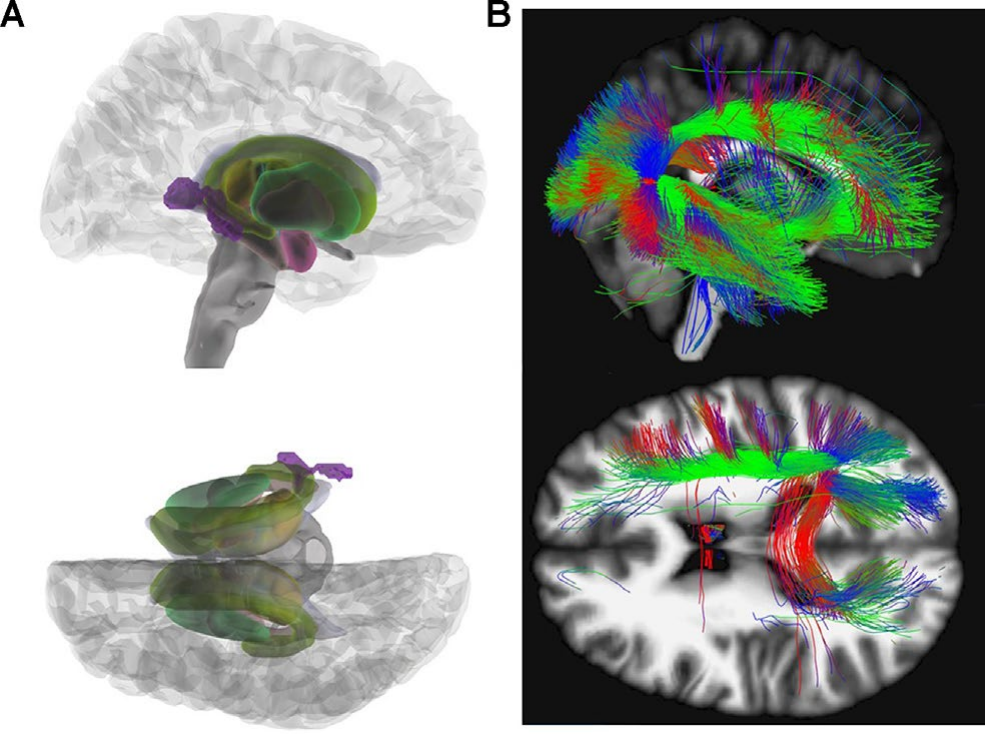
Mean diffusivity (MD). **(A)** Glass brain view of the difference in MD signal depicted in purple between AN and HC individuals in right and top view. Individuals with AN show significantly lower MD values in the posterior thalamic radiation (x = 36, y = −50, z = 8; Z = 4.77) as compared to HC. **(B)** Network involvement of the region with abnormal MD signal. We tracked the connectivity of the ROI in the posterior thalamic radiation (x = 36, y = −50, z = 8) which showed a significantly lower MD in AN to illustrate the network involvement. Red: left – right; green: anterior – posterior; blue: superior – inferior. MD, mean diffusivity; AN, anorexia nervosa; HC, healthy controls; ROI, region of interest.

In the group-wise comparisons (HC versus AN), there was a significant MD decrease in the right posterior thalamic radiation reaching significance on cluster- as well as on peak level (x = 36, y = −50, z = 8; Z = 4.77; k = 493; pFWEpeak = 0.018; pFWEcluster = 0.028). REC and HC showed no significant differences in MD after FWE correction. After correction for effects of depression according to BDI-II (35, 36) no significant results remained. **Figure 2** illustrates our results for MD.

#### Regression Models

No significant correlations neither between FA and BMI nor EDE total score (33) could be detected.

Calculated across all groups, there is a positive correlation between the BMI and the MD in the following areas: the right middle temporal gyrus (x = 60, y = −20, z = −10; Z = 4.95; k = 226; pFWEpeak = 0.008; pFWEcluster = 0.217) and the right lingual gyrus (x = 14, y = −62, z = −3; Z = 4.57; k = 225; pFWEpeak = 0.041; pFWEcluster = 0.219).

There is a negative correlation between the EDE total score (33) and the MD in the left precuneus (x = −10, y = −54, z = 42; Z = 4.83; k = 129; pFWEpeak = 0.014; pFWEcluster = 0.479) and the left inferior frontal gyrus (x = −51, y = 4, z = 30; Z = 4.69; k = 43; pFWEpeak = 0.026; pFWEcluster = 0.870).

## Discussion

This DTI study is so far the largest focusing on differences in FA and MD between adult women with AN in comparison to REC and HC groups. We found a decreased FA in the body of the corpus callosum and a reduced MD in the posterior thalamic radiation in acute AN. Although the REC group showed reduced FA compared with HC, there was no difference in MD detectable after recovery. Our results suggest only partial regeneration of affected WM alterations after recovery from acute AN.

### Fractional Anisotropy

Our result of reduced FA in acute AN adults is in line with most previous studies (12, 14, 15, 21–24, 25, 27, 28). Nevertheless, few studies suggest FA increase (17, 19, 25) in AN or no alterations between AN and HC (18). Apart from often small sample sizes another source of heterogeneity might result from different AN subtypes, differences in duration of illness, age of onset and symptom severity as well as inclusion of patients in different stages of refeeding therapy across the various studies. Further influencing factors are the intake of psychiatric medication and differences in data processing.

Various areas have been reported to be affected by FA alterations in AN patients. In accordance with our results, a recent meta-analysis investigating FA in patients with AN in comparison to HC detected the largest cluster with decreased FA in the corpus callosum (31). Von Schwanenflug et al. (12), Gaudio et al. (15), and Travis et al. (22) found a FA decrease in the same subregion (body) of the corpus callosum. Although these three investigations had studied adolescents, we focused on adults with a longer duration of illness. The corpus callosum facilitates communication between left- and right-sided brain structures (44). The body of the corpus callosum is considered to connect precentral frontal regions and parietal lobes and is involved in several motor, perceptual, and cognitive functions (see **Figure 1**) (45). Altered WM integrity may, therefore, contribute to the distorted body perception in AN (12, 46).

To date, the underlying neurophysiological mechanisms of reduced FA remain unclear. The alterations in WM microstructure could either be a premorbid trait marker or result from malnutrition (47). Axon density, fiber geometry, and myelination are proposed to contribute to the DTI-signal (48). A tendency for larger diameter axons was suggested for medial and posterior cross-sections of the corpus callosum (49, 50). Previous investigations propose that axons of larger diameter tend to have thicker myelin sheets with higher concentration of lipids (51). This may render the body of the corpus callosum more susceptible to myelin loss due to lipolytic mechanisms following malnutrition (52).

### Mean Diffusivity

MD computes the average diffusion irrespective of directionality. It is sensitive to myelin changes as well as variations in intra/extra cellular spaces (53). Previous studies focusing on MD are heterogeneous with some reporting increased MD in adults (23, 24) others no MD differences (15, 18) or decreased MD in adolescents with AN (17, 24). We found an MD decrease in the posterior thalamic radiation, an area where a decreased FA in AN patients has already been described (27). Fibers of the posterior thalamic radiation project into the occipital, the temporal, and parietal cortex and connect to cortical regions involved in processing of the body image (see **Figure 2**) (27).

It was supposed that MD decrease might result from dehydration in AN, which could potentially limit water diffusion (24). However, previous AN studies assessed hydration by measuring urine specific gravity prior to scanning but found no evidence of dehydration or hyperhydration in their sample (12, 18). It is not yet clarified whether urine specific gravity may sufficiently reflect the hydration status (54).

### Recovery

We detected FA decrease in the corpus callosum in REC in comparison to HC, whereas no MD differences were detectable. Studies investigating AN patients after recovery are scarce, and findings are inconsistent. In line with our finding, two cross-sectional studies reported FA differences between REC and HC (20, 27) and two longitudinal investigations suggested only partial rehabilitation of WM after weight restoration (17, 19). In contrast to our results, four studies found no differences in microstructural integrity between REC and HC (12, 13, 18, 26). The discrepant results might be due to differences in sample characteristics, including definition of recovery and analytical approach. Therefore, future studies should be carefully controlled and include well-defined samples (55, 56).

Our results of FA decrease in the corpus callosum after prolonged weight restoration and absence of AN symptoms indicate that complete recovery may be a long-lasting process, i.e., the duration of recovery of our sample was too short to detect complete reversibility. Furthermore, a parallel study of GM and WM did not detect differences between REC and HC (40). However, due to our strict inclusion criteria, it likely represents a permanent “scar” of the acute disease process. Alternatively, the FA decrease in AN and REC could also be regarded as a persisting premorbid trait, i.e., a neural endophenotype. To clarify this hypothesis, an investigation of disease-free siblings and a longitudinal study design might shed light on the issue.

The detection of no differences in MD between the REC and HC groups supports the hypothesis that a reduced intensity of diffusion is reversible following weight restoration.

### Regression Models

The positive correlation between MD and BMI is in line with the assumption that MD alterations in AN patients are associated with dynamic processes. As underlying factors dehydration or malnutrition would be conceivable (57).

We detected a negative correlation between the EDE total score (33) and the MD in the left precuneus and the inferior frontal gyrus. In the precuneus, a region associated with self-processing and episodic autobiographic memory retrieval (58), a diminished GM in AN has already been detected (59). The left inferior frontal gyrus is suggested to be involved in impulsivity regulation (60).

No correlation neither between FA and BMI nor between FA and EDE total score (33) could be found. This is in line with the meta-analysis by Barona et al. (31) who reported no association between BMI- and AN-related FA reductions. One possible explanation could be that, in contrast to the MD, the FA is not primarily weight-dependent and, therefore, not state-related.

### Methodological Issues and Limitations

Strengths of the current study are the well-defined AN sample, strict definition of recovery criteria and the inclusion of relevant covariates (age, TIV, depression).

The sample size compares well to other studies in the field. In fact, so far this is the largest DTI study in adults with AN, REC, and HC. Still, larger samples sizes might have allowed the detection of possible more subtle differences. Another limitation of our study is that we did not check for the hydration status of participants which might have helped with the interpretation of the MD findings. Moreover, weight restoration before scanning was not specifically evaluated; however, we do not assume relevant weight increase as patients were largely outpatients seeking treatment or at the beginning of inpatient treatment.

Our MD results did not survive a correction for the measures of depression when adding the BDI-II scores as a covariate to the statistical analysis. Since AN symptoms and BDI scores were highly correlated, we cannot disentangle their influences and, therefore, cannot rule out any confounding effects arising from depression. Similar effects of comorbid depressive symptoms are found in other psychiatric disorders, too, and are difficult to disentangle, which we discussed previously (40, 61). In contrast, following correction for the effect of depression, FA signals of REC and HC still differed significantly from each other. Therefore, it is unlikely that the reduced FA in AN is driven solely by effects of depression. However, it is important to recognize that depressive symptoms may be regarded as the sequel of malnutrition in AN (62). The chronicity of the illness itself can induce depressive symptoms as in most chronic diseases (63). Our study sample had a relatively long duration of illness and might, therefore, show more pronounced depressive symptoms as compared to other studies. The only way to disentangle AN-only and depressive effects would be to recruit a depressive control group. However, reversely, even in this setting, malnutrition effects cannot be excluded due to loss of appetite in depressive patients.

Results in previous studies may have been biased by ventricular enlargements in AN (13). Recent investigations showed that especially differences in fornix FA between AN and HC disappeared after correction for cerebrospinal fluid partial volume effects (64). Therefore, it is possible that findings of reduced FA in AN, especially in areas close to ventricles, are biased (13). Longitudinal studies could provide more insight into whether complete reversibility is possible after a longer period of recovery.

### Summary

In this study of women with acute AN, control subjects, and recovered patients, we could confirm impairments of WM integrity in acute AN. Differences in FA were detectable between the REC and HC groups, whereas there were no alterations between REC and HC concerning MD. Thus, impairments of FA measures possibly reflecting disturbed brain connectivity may either be seen as a trait marker of AN or a “connectivity scar,” whereas reduced MD measure probably represents state markers of the acute state of AN. The underlying neurobiological mechanisms are not yet clarified. To get more information about the existence of pre-morbid markers, studies on participants at risk for anorexia need to be conducted. In future studies, it is important to follow precisely defined guidelines and to include carefully selected samples (55, 56).

## Ethics Statement

The present study was approved by the ethics committee of the University Medical Center Freiburg (Approval ID: 520/13)

## Author Contributions

KN, SM, and AJ performed the data analysis and wrote the manuscript. AJ, LTvE, AZ, and SM designed the study, and AJ is the principal investigator of the study funded by DFG-Grant JO 744-2/1. All authors were crucially involved in the theoretical discussion and the preparation of the manuscript. All authors have made substantial contributions to conception and design, acquisition of data or analysis, and interpretation of data. All authors read and approved the final version of the manuscript. They agreed to be accountable for all aspects of the work.

## Funding

The article processing charge was funded by the German Research Foundation (DFG) and the University of Freiburg in the funding program Open Access Publishing. Financial support: Part of project DFG JO 744-2/1.

## Conflict of Interest Statement

LE has been involved in advisory boards and lectures, or has received travel grants within the last 4 years from Eli Lilly, Janssen-Cilag, Novartis, Shire, UCB, GSK, Servier, Janssen, and Cyberonics.

The remaining authors declare that the research was conducted in the absence of any commercial or financial relationships that could be construed as a potential conflict of interest.
